# PAPSS2 Promotes Alkaline Phosphates Activity and Mineralization of Osteoblastic MC3T3-E1 Cells by Crosstalk and Smads Signal Pathways

**DOI:** 10.1371/journal.pone.0043475

**Published:** 2012-08-16

**Authors:** Weizhuo Wang, Fang Li, Kunzheng Wang, Bin Cheng, Xiong Guo

**Affiliations:** 1 Department of Orthopedics Surgery, the Second Affiliated Hospital, College of Medicine, Xi'an Jiaotong University, Xi'an, Shaanxi, People's Republic of China; 2 Department of Prosthodontics, School of Stomatology, The Fourth Military University, Xi'an, Shaanxi, People's Republic of China; 3 Faculty of Public Health, College of Medicine, Key Laboratory of Environment and Gene Related Diseases of Ministry Education, Xi'an Jiaotong University, Xi'an, Shaanxi, People's Republic of China; Oklahoma State University, United States of America

## Abstract

Several studies have indicated that PAPSS2 (3′-phosphoadenosine-5′-phosphosulfate synthetase 2) activity is important to normal skeletal development. Mouse PAPSS2 is predominantly expressed during the formation of the skeleton and cartilaginous elements of the mouse embryo and in newborn mice. However, the role and mechanism of PAPSS2 in bone formation remains largely unidentified. By analyzing the expression pattern of the PAPSS2 gene, we have found that PAPSS2 is expressed in bone tissue and bone formation. PAPSS2 transcripts increase during osteoblast differentiation and are in less level in RANKL-induced osteoclast like cells. By using lentivirus-mediated RNA interference (RNAi) technology, we knocked down PAPSS2 expression in MC3T3-E1 osteoblast. Silencing of PAPSS2 expression significantly decreases ALP activity and cell mineralization, inhibits expression of osteoblast marker osteopontin (OPN) and collagen I. Conversely, overexpression of PAPSS2 promotes the MC3T3-E1 to differentiate into osteoblast and mineralization. Moreover, compared to that in the control cells, the mRNA level and protein expression of phosphorylated Smad 2/3, which is a key transcriptional factor in the Smad osteoblast differentiation pathway, showed significant decreases in PAPSS2-silenced cells and increases in PAPSS2-overexpression cells. These results suggest that PAPSS2 might regulate osteoblast ALP activity and cell mineralization, probably through Smads signal pathways.

## Introduction

Bone is a mineralized tissue that underlies multiple mechanical and metabolic functions of the skeleton [1], [2]. Formation and maintenance of bone tissue are regulated in a sophisticated fashion by bone-forming osteoblasts and bone-resorbing osteoclasts. Many skeletal diseases, such as osteoporosis, Kashin-Beck disease, Paget's disease of the bone, rheumatoid arthritis, and bone metastases all arise from an imbalance in the relative activities of osteoblasts and osteoclasts [3]. The proliferation and differentiation of those two cell types are controlled by various local growth factors, cytokines produced, in the bone and by systemic enzymes. The presence of PAPS (3′-phosphoadenosine-5′-phosphosulfate) is a prerequisite for catalytic efficiency in all sulfation reactions. In humans, PAPS is synthesized from ATP and SO_4_^2−^ by two isoforms of PAPS synthetase (PAPSS): PAPSS1 and PAPSS2. The PAPSS2 is one of the principal enzymes required for the sulfation of extracellular matrix molecules in bone formation and other tissues [4]–[6]. A truncation mutation of the human PAPSS2 gene was reported in a Pakistani family suffering from a novel form of spondyloepimetaphyseal dysplasia (SEMD) [7], [8]. A homozygous PAPSS2 mutation (S475X) was identified in another large Pakistani family affected by SEMD, Pakistani type [8], [9]. This manifested as disproportionately short stature with short, bowed lower limbs, enlarged knee joints, kyphoscoliosis, and generalized brachydactyly [7]. Chondroitin 6-O-sulfotransferase requires PAPS for catalytic activity, and the abnormal PAPSS2 gene that encodes chondroitin 6-O-sulfotransferase can cause SEMD, Omani type [10]. Humans lacking normal PAPSS2 activity exhibit long bone shortening and bowing, and also show degenerative joint disease, including evidence of knee joint arthrosis. Given the premature development of degenerative knee joint disease in the mutant mice and other similarities between SEMD mice and human lacking normal PAPSS2 activity, it has been proposed that this mutant represents a model of human PAPSS2 deficiency-associated arthrosis [11].

One of our previous studies included a microarray analysis that showed some correlation between PAPSS2 genes in individuals afflicted with endemic knee osteoarthritis and those with Kashin-Beck disease exhibiting shortened long bones and enlarged knee and finger joints [12], [13]. Some features of the bone phenotype in the endemic osteoarthritis patient resemble those observed in SEMD, excepting that the changes in the bone changes were milder for our patients and the changes in the epiphyseal plates of the long bones and metaphyseal changes were detectable in our patients [14], [15]. However, the absence of the pubertal growth spurt characteristic of in Kashin-beck disease, compromised final height, and the increased ratio of sitting height to standing height are indicative of bone dysplasia [14], [16].

These observations suggest that PAPSS2 may participate in the control of important physiological processes in bone and cartilage, such as collagen fibrillogenesis and/or matrix calcification and mineralization. However, the role of PAPSS2 in bone development and formation and the mechanisms underlying this role remain largely unidentified. In order to better understand the role of PAPSS2 in various biochemical pathways, its molecular biology, biochemistry, structure, and function must be thoroughly studied. In this study, evaluated role of PAPSS2 in osteoblasts by detailing the function of the gene in the formation of a mineralization-competent bone matrix through activated pathway [5], [17].

## Results

### Expression of PAPSS2 mRNA in bone and role in osteoblast differentiation

**Figure 1:**
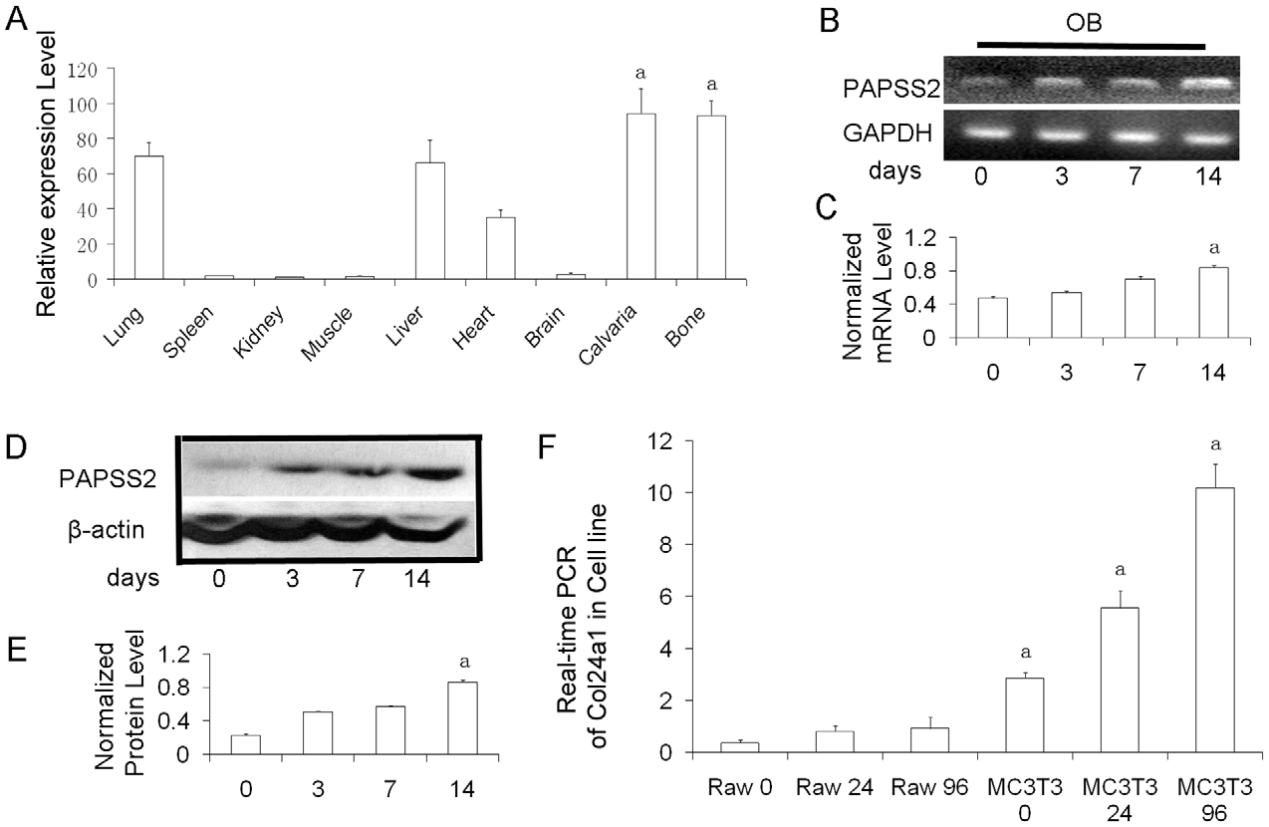
PAPSS2 is prominently expressed in osteoblasts derived from MC3T3 cells stimulated by ascorbic acid and β-glycerol-phosphate (OS media). (A) Tissue distribution of PAPSS2 mRNA expression in lean control mice. Total RNA was extracted from the indicated tissues, reverse transcribed, and assessed using quantitative real-time PCR. PAPSS2 mRNA expression was calculated relative to the expression of B2m mRNA, and values were expressed relative to the kidney level. (^a^ *P*<0.05 between calvaria/bone and the other tissues) (B) RT-PCR analysis of PAPSS2 mRNA in MC3T3, for 0, 3, 7, and 14 days, showing that PAPSS2 is expressed in OB. (C) Normalized mRNA level of *B*. (^a^ *p*<0.05 compared with 0 days) (D) Western blot analysis of PAPSS2 protein expression in OB derived from MC3T3 cells induced with OS media for 0, 3, 7, and 14 days. Equivalent amounts (20 μg) of total protein were loaded in each lane. (E) Quantification of protein levels from immunoblots, as in *D*. The protein levels of PAPSS2 were normalized to GAPDH. The levels of PAPSS2 were gradually increased in timeline. (^a^ *P*<0.05 compared with 0 day) (F) Real-time PCR analysis of PAPSS2 expression pattern during differentiation of osteoclast and osteoblasts. RAW264.7 (Raw) cells were induced with 10 ng/ml of RANKL and 20 ng/ml M-CSF for 0, 24, and 96 hours for osteoclastogenesis; and MC3T3-E1 clone 4 cells were induced with OS media for 0, 7, and 21 days, respectively. (^a^ *P*<0.05 between raw and MC3T3 for 0, 7, 21 days). The results showed that PAPSS2 is specifically and highly expressed in both pre-osteoblasts and osteoblast.

We assessed the expression of PAPSS2 mRNA in various organs and tissues from lean control mice using quantitative real-time PCR. We found that the expression of PAPSS2 was very high in the bones, liver, and lungs and moderate in the heart. The mRNA level was extremely low in the spleen, muscle, kidneys, and brain (Fig. 1A). The housekeeping gene murine B2m was expressed at an almost constant level in the organs and tissues examined, validating the present real-time PCR method for measurement of PAPSS2 mRNA. These findings were also consistent with those of previous reports [18]. PAPSS2 was more highly expressed in human osteoblasts than in human osteoclasts (Figs. 1B, C, F). These results were confirmed by Western blot analysis in MC3T3 cell lines (Figs. 1D, E). Our results showed that PAPSS2 was prominently expressed in authentic mouse osteoblasts exposed to OS media at both the mRNA and protein levels. This suggests that PAPSS2 might play important role in osteoblast differentiation *in vitro*.

### Silence of PAPSS2 attenuates mineralization and the ALP activity

**Figure 2:**
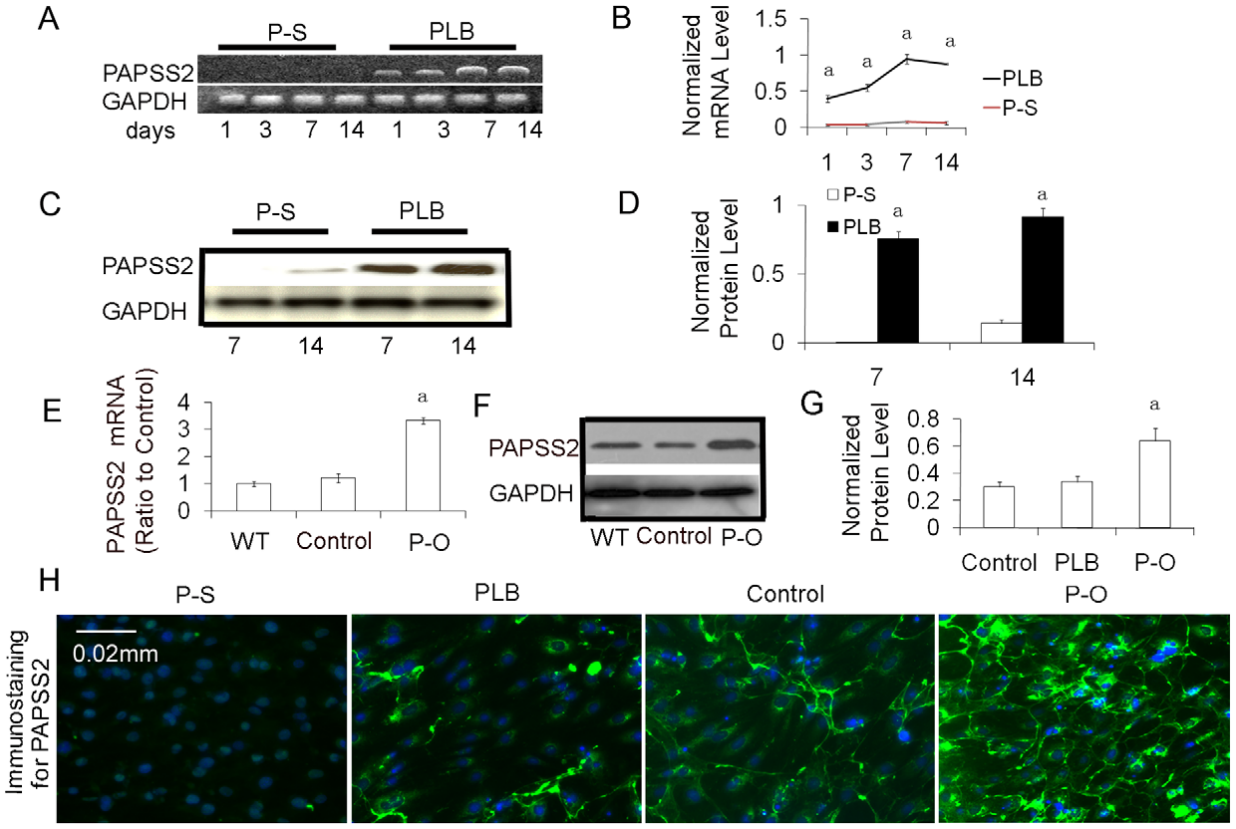
Silencing of PAPSS2(P–S) with lentivirus transfection block PAPSS2 expression and overexpression of PAPSS2 (P–O) with adenovirus system significantly increases the PAPSS2 expression. (A) RT-PCR analysis. MC3T3-E1 clone 4 cells were infected with plenti-shRNA PAPSS2 (P–S) or plenti-scrambled shRNA (PLB) viruses for 48 hours and then induced with OS media for 0, 3, 7, and 14 days. The transcription level of PAPSS2 gradually increased in PLB cells (lanes 1, 2, 3, and 4) and were much lower or undetectable in P–S cells (lanes 4, 5, 6, and 7). (B) Values in A are normalized to GAPDH levels. (^a^ *P*<0.05 between P–O and PLB for 1, 3, 7, and 14 days). (C) Western blot analysis of PAPSS2 protein expression. The cells were treated as described in A. (D) Quantitative analysis of protein levels from immunoblots, as in C. The protein levels of PAPSS2 were normalized to GAPDH. (^a^ *P*<0.05 between P–O and PLB for 7 and 14 days). The level of PAPSS2 in the silenced group was 426.6 times (7 d) and 6.6 times (14 d) lower than that of the control. (E) Real-time PCR analysis of PAPSS2 expression in MC3T3-E1 cells infected with pBMN-I-GFP (control) or pBMN-PAPSS2 (P–O) for 24 hours. It was then induced with OS media for 14 days. The signals were significantly increased in P–O group compared with that in control group (^a^ *P*<0.05 between P–O and the control). (F) Western blot analysis of PAPSS2 protein expression after cells were treated as described in E. (G) Quantitative analysis of protein levels from immunblots, as in F. The protein levels of PAPSS2 were normalized to GAPDH. The level of PAPSS2 in the silenced group was 1.9 times (14 days) higher than that of the control. (^a^ *P*<0.05 between P–O and the control) (H) Immunofluorescence staining revealed that the expression of PAPSS2 was blocked after infection with P–S and overexpression in P–O cells relative to cells without virus transfection (control) and to cells without P–O after 14 days of induction with OS media. The cell nuclei were stained using DAPI (blue) to identify the number of cells.

To demonstrate the importance of PAPSS2 function in osteoblast differentiation, we used lentivirus-mediated RNA interference (RNAi) technology to silence (knock down) PAPSS2 expression in MC3T3-E1 exposed to OS media [19]. After stable transfection with constructs encoding small interfering RNA (siRNA) targeting PAPSS2, we confirmed the effects of RNAi and performed RT-PCR (Figs. 2A, B), Western blot analysis (Fig. 2C, D), and immunofluorescent staining (Fig. 2H) to assess silencing efficiency. As shown in Fig. 2A, PAPSS2 mRNA transcription and protein expression were silenced in stably transfected PAPSS2-silenced cell lines from 3, 7, and 14 days, as indicated by comparison to control cells transfected with the empty vector. In contrast to wild-type cells, we found that the cells infected with PAPSS2-silencing lentivirus contained undetectable levels of PAPSS2 protein at 7 days after transfection (Fig. 2C). To determine the effects of PAPSS2 RNAi on osteoblast gene expression, we also examined the expression of PAPSS2 using immunofluorescent staining 7 days after infection. As shown in Fig. 2E, PAPSS2 silencing blocked PAPSS2 protein expression. These results were consistent with those of RT-PCR and Western blot analyses.

**Figure 3:**
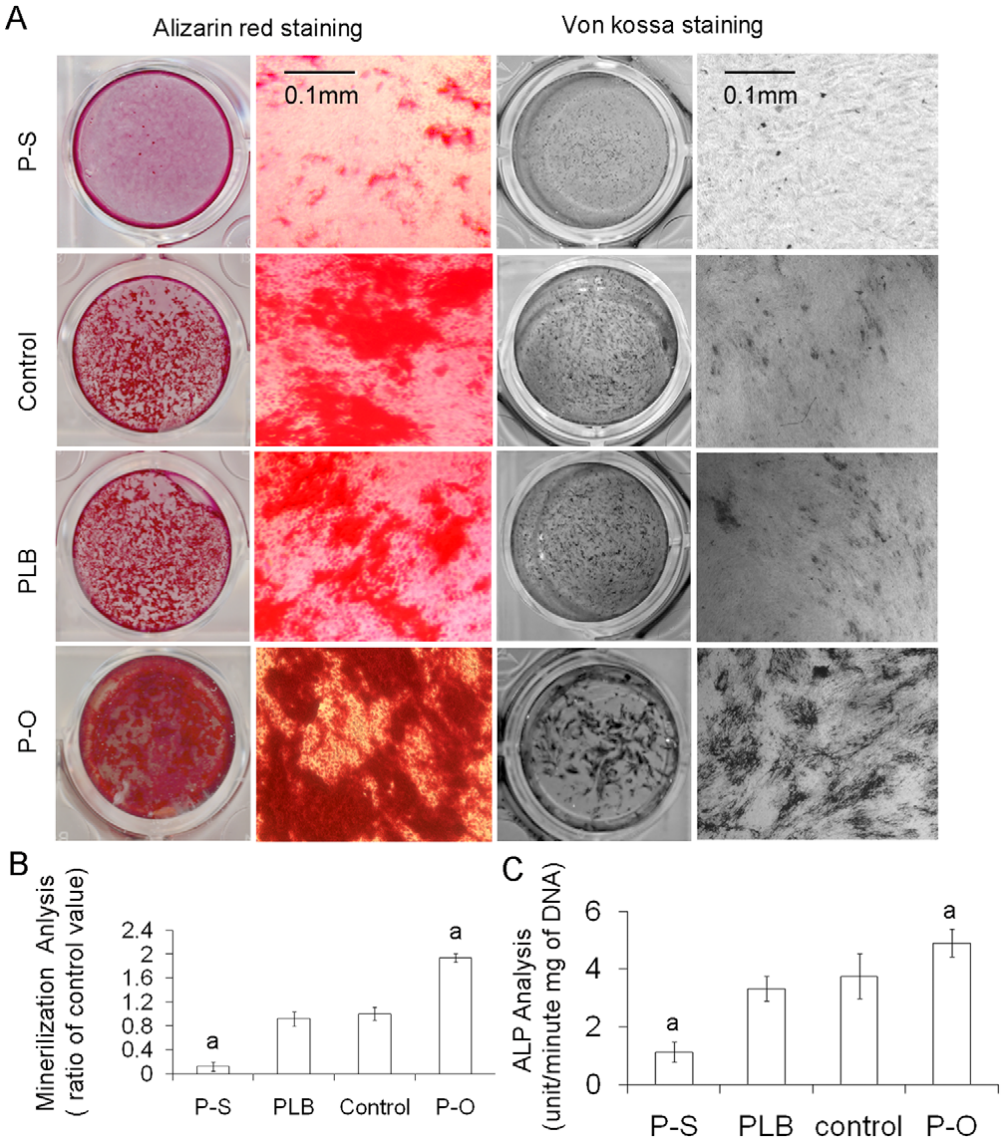
Influence of PAPSS2 on ALP activity and mineralization in MC3T3-E1 cells. MC3T3-E1 cells were infected with pBMN-I-GFP (control) or pBMN-PAPSS2 (P–O) for 24 hours, plenti-shRNA PAPSS2 (P–S) or plenti-scrambled shRNA (PLB) viruses for 48 hours and then induced with OS media for 7 and 14 days. (A) Effects of P–O on mineralization of MC3T3-E1 cells. The calcium deposits in the mineralized matrix were analyzed with the Aliralin red staining and Von Kossa staining. (B) Quantitative analysis of mineralization from Alizarin red staining. Each value is expressed as a ratio of the value in PAPSS2-silenced cells to that in PLB transfected cells. A *P*<0.05 was observed between P–O and the control, P–S, and the PLB. (C) Effects of P–O on ALP activity in OS media induced by MC3T3-E1 cells. Each bar is expressed as the mean± SD (unit/min.mg DNA) of four determinations. a *P<*0.05 in the value between P–O and control group, P–S and the PLB was observed.

ALP activity and mineralization are important to bone formation, as are matrix proteins. ALP hydrolyzes pyrophosphate and provides inorganic phosphate to promote mineralization in osteoblasts. We determined whether PAPSS2 would affect ALP activity and mineralization in MC3T3-E1 cells. ALP activity was evaluated biochemically. Mineralization was examined using Alizarin red staining, Kossa staining, and a quantitative assay of mineralization based on Alizarin red staining (Figs. 3A, B). As shown in Fig. 3A, calcium deposits stained with von Kossa and Alizarin red S consistently showed that both PAPSS2 silencing and PAPSS2 overexpression significantly decreased ALP activity and cell mineralization. ALP activity was measured at 7 days and was found to be significantly different in the silencing and overexpression groups (*P<*0.05) (Fig. 3C). These data indicate that PAPSS2 silencing in MC3T3-E1 cells can block ALP activity and mineralization in osteoblastic cells.

### Effects of overexpression of PAPSS2 on ALP activity and mineralization of osteoblastic cells

To gain further insight into the molecular mechanisms of PAPSS2 function in osteoblast differentiation, we overexpressed PAPSS2 in MC3T3-E1 clone 4 induced with OS media [19]. The PBMN-I-GFP (vector-only control) and PBMN-I-GFP-PAPSS2 were transfected into the Phoenix ecotropic packaging cell line. After stable transfection with constructs, we confirmed the effect of RNAi and performed real-time RT-PCR (Fig. 2E), Western blotting (Figs. 2F, G), and immunofluorescent staining (Fig. 2H) to assess overexpression. Unlike wild-type cells, we found that the expression of PAPSS2 protein increased in cells infected with virus. As shown in Figs. 2E–G, the overexpression of PAPSS2 both on the mRNA and protein levels was detectable in cell lines stably transfected with PBMN-I-GFP-PAPSS2 but not in cells transfected with empty vector. As shown in Fig/3A, mineralization signals were detected in transfected MC3T3-E1 cells. Different signals were observed in cells transfected with empty vector. ALP activity and cell mineralization were both much higher in cells overexpressing PAPSS2 than in cells transfected with empty vector at both 7 and 14 days after induction by OS media. Quantization of mineralization with Alizarin red staining indicated that the mineralization of the overexpression group and silencing group were significantly different from those of the control (*P*<0.05). As demonstrated in Fig. 3C, ALP activity of PAPSS2 overexpression and silence was significantly different (*P<*0.05) from the value of control group after incubation for 7 d. PAPSS2 overexpression-transfected cells had much higher ALP activity and mineralization than cells transfected with empty vector. These data indicate that PAPSS2 promotes ALP activity and mineralization of osteoblastic cells.

### Effects of PAPSS2 gene on the expression of osteoblast-specific marker

**Figure 4:**
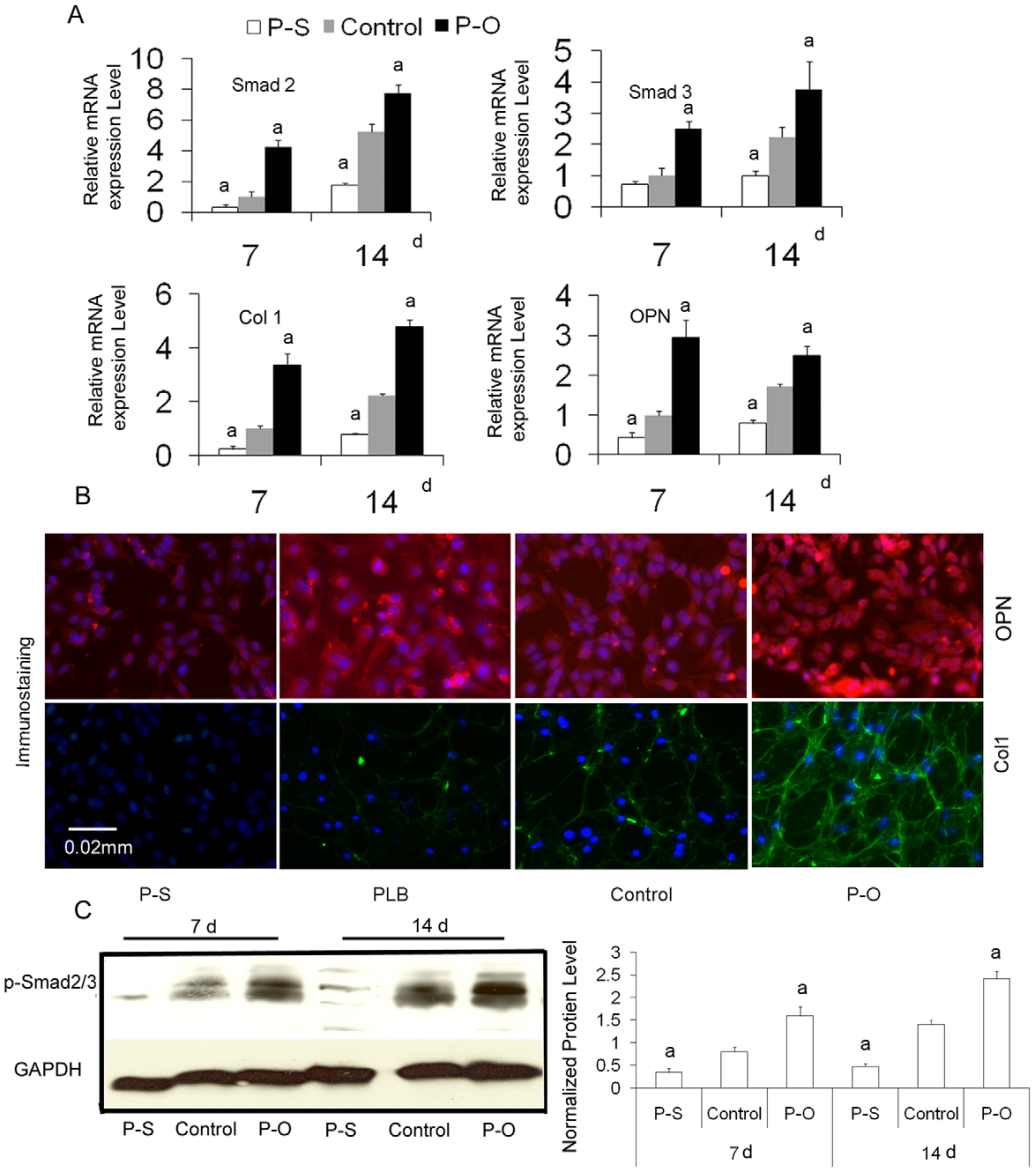
PAPSS2 silencing (P–S) suppresses expression of osteoblast marker genes and crosstalk with Smads pathway. MC3T3-E1 cells were infected with pBMN-I-GFP (Control) or pBMN-PAPSS2 (P–O) for 24 hours, plenti-shRNA PAPSS2 (P–S) or plenti-scrambled shRNA (PLB) viruses for 48 hours and then induced with OS media for 7 and 14 days. (A) Real-time RT-PCR analysis of Samd2, Samd3, Col I, and OPN mRNA in MC3T3-E1 cells induced with OS media for 7 and 14 days, showing that the expression of Samd2, Smad3, Col I, and OPN was attenuated in P–S cells compare with PLB cells. Conversely, the expression levels of Samd2, Smad3, Col I, and OPN were higher in P–O cells than in control cells (a *P<*0.05). (B) Immunofluorescence of MC3T3-E1 cells revealed that the expression of type 1 collagen (Col1, green fluorescence) and osteopontin (OPN, red fluorescence) was lower in P–S cells than in PLB cells. The expression levels of Col I and OPN were higher in P–O cells than in control cells at 14 days. The cell nuclei were stained using DAPI (blue) to identify the number of cells. (C) Western blot analysis of p-Smad2/3 protein expression. Cells were prepared as A. A marked difference was observed in the levels of p-Smad2/3 between P–O and the control groups and between P–S and the PLB groups. (D) The protein levels of p-Smad2/3 in G were normalized to GAPDH. The levels of p-Smad2/3 in PAPSS2-overexpressed and -silenced cells induced with OS media for 7 days were 2.0 and 0.4 times that in the control group and at14 days they were 1.7 and 0.3 times that in the control group. *P<*0.05 was observed between P–O and control group, P–S and control group were observed.

The osteoblast-associated molecules Col I and OPN are considered osteoblast differentiation markers because their expression levels increase during osteoblast differentiation [1], [20]. To determine the effects of PAPSS2 overexpression and silencing on osteoblast differentiation in MC3T3-E1 cells, mRNA levels of Col1 and OPN were examined using quantitative real-time PCR. The mRNA levels of Col1 and OPN were changed after treatment, which lasted 7 days after infection. The signals of Col1 and OPN were stronger than control values in cells overexpressing PAPSS2 and weaker than control values in PAPSS2-silenced cells (Fig. 4A). The protein expression of Col1 and OPN (Fig. 4C, D) was confirmed by immunofluorescence of MC3T3 cells. Results showed that the expression of type 1 collagen and OPN was attenuated in PAPSS2-silenced cells and increased in PAPSS2-overexpressing cells (Fig. 4B). These results suggest that PAPSS2 can facilitate osteoblast differentiation by regulating the expression of Col1 and OPN.

### Modulation of the Smad pathway in the transfected osteoblastic cells

The Smad family proteins are critical components of the TGF signaling pathways. Smad signaling is critical to the regulation of osteoblast differentiation. Smad signal expression is indicative of the function of the PAPSS2 pathway. To determine the mechanism by which PAPSS2 enhances ALP activity and mineralization, we determined whether PAPSS2 silencing and overexpression would affect the protein or mRNA levels of p-Smad2/3 in MC3T3-E1 cells using Western blot analysis and real-time RT-PCR, respectively. Real-time RT-PCR analysis of Smad2 and Smad3 showed that PAPSS2 silencing significantly reduced the mRNA levels of Smad2 and Smad3 in osteoblasts derived from MC3T3-E1 cells (Fig. 4A). Western blot analysis of p-Smad2/3 protein expression in empty vectors and in transfected MC3T3-E1 cells subjected to PAPSS2 silencing and overexpression showed that protein levels of Smad2/3 in these cells differed from that of the control groups (Fig. 4C). The levels of p-Smad2/3 in the overexpression group and in the silencing group, which were stimulated with OS media for 7 d, were 2.0 and 0.4 times that of control cells. For cells stimulated for 14 d, they were 1.7 and 0.3 times of that of control cells (Figs. 4C, D). Transfected cells subjected to PAPSS2 silencing showed lower levels of Smad 3 than cells transfected with empty vector under real-time RT-PCR analysis. These data indicate that PAPSS2 might stimulate the expression of bone matrix proteins such as Smad 3 in osteoblastic cells. We demonstrated that the involvement of PAPSS2 in the Smad signaling pathway plays a role in the fast effect of osteoblast differentiation.

## Discussion

During skeletogenesis, bone is formed in two different ways, intramembranous ossification and endochondral ossification. In intramembranous ossification, osteogenesis occurs within the condensed mesenchymal cells. This type of ossification produces the flat bones and the additional bone. In endochondral ossification, mesenchymal cells first condense to form a cartilage model. Then bone slowly forms to replace this cartilage. This type of ossification produces most of the bones of the body, including the axial and appendicular skeletons.

Inactivating mutations for PAPSS2 are associated with severe inherited developmental skeletal disorders, Kanshin Beck disease, spondyloepimetaphyseal dysplasia in humans, and brachymorphism in mice [8], [12]. The deficiency of PAPSS2 results in osteochondrodysplasias. Osteoechondrodysplasias are a genetically heterogeneous group of disorders that affect skeletal development, linear growth, and the maintenance of cartilage and bone. A large inbred family of mice with a distinct form of recessively inherited, spondyloepimetaphyseal dysplasia (SEMD) was examined. Their condition was attributed to a PAPSS2 isoform located in the chromosome region of 10q23–24 [21]. Diastrophic dysplasia is an autosomal recessive osteochondrodysplasia that constitutes disorders of the skeletal system that can have defect on growth or bone density (the physical characteristics include, short limb stature, cleft palate, generalized dysplasia of joints, hitchhiker thumb etc.). The abnormality is due to deficiency in the intracellular sulfate, which then leads to under sulfated proteoglycan [22]. Sequence polymorphisms of PAPSS2 with reference to osteoarthritis were investigated by Ikeda et al. [11], [23], [24]. OA is a musculoskeletal disorder characterized by degeneration of articular cartilage. The SNP analysis of the PAPSS2 from certain Japanese populations suffering from knee OA showed some correlation with PAPSS2. Differential distributions of the two isoforms of PAPS synthase were manifested only in tissues affected by OA [24].

MC3T3-E1 pre-osteoblastic cells undergo differentiation and formation of a mineralizing matrix. These cell cultures are frequently used to investigate the role of specific genes in osteoblast differentiation. In this study, knocking down PAPSS2 with siRNA or by viral transduction with shRNA showed that PAPSS2 plays an important role in promoting osteoblast differentiation. When PAPSS2 is depleted, multiple markers of osteoblast differentiation were significantly reduced. This included mRNA levels of collagen Ι and OPN. PAPSS2 siRNA significantly reduced the up-regulation of alkaline phosphatase activity that occurred as a result of osteoblast differentiation. These results are unlikely to be due to global changes because PAPSS2 knockdown did not affect that overall protein or total RNA levels per well (data not shown). OPN has been implicated as an important factor in bone remodeling [25]. The organic part of bone is about 20% of the dry weight, and counts in, other than OPN, collagen type I, osteocalcin, osteonectin, bone sialo protein and alkaline phosphatase. Collagen type I accounts for 90% of the protein mass. Osteoblastic differentiation is accompanied by increased expression of the prominent osteoblastic marker, OPN. Different cell types may differ in their methods of regulating the OPN gene. OPN expression in bone occurs predominantly in osteoblasts, osteocyctes, and osteoclasts [26]. PAPSS2 knockdown reduced OPN mRNA levels, sharply, indicating that PAPSS2 acts upstream of OPN induction. It is possible that the biochemical mechanism of PAPSS2 affects osteoblast differentiation. This would include direct targeting of the OPN gene, which was confirmed in immunostaining assays. These assays have indicated that PAPSS2 targets the OPN promoter directly. The dynamics of this interaction correlates specifically with induction of differentiation. The reduction of OPN mRNA levels implies that direct targeting of downstream osteogenic genes by OPN is impaired alongside depletion of PAPSS2. These results do not rule out the possibility that there are multiple mechanisms by which PAPSS2 directly or indirectly modulates OPN activity and expression of osteogenic genes, but they establish an early, direct, and essential role for PAPSS2 in osteoblast differentiation. The other genes that were modulated by PAPSS2 knockdown, which includes transcription factors, enzymes associated with differentiated function, bone morphogenetic proteins, and extracellular matrix proteins, need be understood more deeply.

TGF/Smads is produced by osteoblasts and appears to regulate bone metabolism in various ways, including skeletal development and bone remodeling [18], [27], [28]. Smad3, a critical component of the Smads signaling pathway, plays an important role in the regulation of bone formation [28]–[34]. Smad3 has been shown to bind to the OPN promoter, on which it acts as a sequence-specific activator [35]. Smad2 mediates the signal of the TGF-β, and thus regulates many cellular processes, such as cell proliferation, apoptosis, and differentiation. This protein is recruited to the TGF-beta receptors through its interaction with the Smad anchor for receptor activation protein. The phosphorylation induces the dissociation of this protein from the Smad anchor for receptor activation and its association with other Smad family members. The association with Smad2/3 is important to the translocation of this protein into the cell nucleus, where it binds to target promoters and forms a transcription repressor complex with other cofactors. This protein can also be phosphorylated by activin type 1 receptor kinase, and it mediates the signal from the activin. Alternatively spliced transcript variants encoding the same protein have been observed [36]. Like other Smads, Smad2 and Smad3 play roles in the transmission of extracellular signals from ligands of the TGF-β superfamily of growth factors into the cell nucleus [37]. Binding of a subgroup of TGF-β superfamily ligands to extracellular receptors triggers the phosphorylation of Smad2 at a serine-serine-methionine-serine motif at its extreme C-terminus [33], [38], [39]. Smad2/3 is a key transcriptional factor in osteoblast differentiation, as indicated by its relationship with PAPSS2. Our results suggest that, Smad signal pathways in osteoblast are at least partially mediated by PAPSS2.

This study demonstrated the regulatory mechanism of PAPSS2 acts during osteoblast differentiation and mineralization, which is one of principal issues remaining to be elucidated in the control of bone formation for better understanding of pathogenesis of osteoporosis and for the development of therapeutic procedures. In conclusion, these results indicate that PAPSS2 plays an essential role in Smads signaling pathways during osteoblast differentiation. Ongoing research in this laboratory will determine the mechanism by which PAPSS2 affects collagen assembly during bone formation and function, which transcription factors or growth factors regulate PAPSS2 gene expression, synthesis, accumulation, and protein degradation, and the manner in which PAPSS2 regulates TGF pathways.

## Materials and Methods

### Cells and cell culture

This study was approved by the Animal Experiment Committee of Xian Jiaotong University. MC3T3-E1 cell line, which is an osteoblast line derived from murine calvaria that differentiates in culture, and RAW264.7, which is a mouse monocytic cell line, and ecotropic Phoenix packaging cells were obtained from American Type Culture Collection(ATCC, Bethesda, MD, U.S.) [40]. The cells were seeded at a density of 1×10^4^ cells/cm^2^ and maintained in growth medium consisting of a-modified Eagle's medium plus 10% fetal bovine serum (FBS, Gibco), 100 U/ml penicillin, and 100 mg/ml streptomycin in a humidified atmosphere containing 5% CO_2_ at 37°C. They were passaged every 5–7 days and not used beyond passage 20. For osteoblast differentiation, the MC3T3-E1 clone 4 cells were induced with osteogenic medium (OS media), specifically, the growth medium containing 50 μg/ml ascorbic acid and 10 mM β-glycerol-phosphate. For osteoclast differentiation, RAW264.7 cells were induced with 10 ng/ml RANKL and 20 ng/ml M-CSF.

### Reverse Transcription and Real-Time PCR Analysis

Total RNA from mouse tissues (lung, spleen, kidney, muscle, liver, heart, brain, calvaria, bone) and from the OS media-induced MC3T3-E1 clone 4 cells, or RANKL/M-CSF induced RAW264.7 cells was isolated using Trizol reagent (Invitrogen). A two-step reverse transcription real-time PCR (Invitrogen) was used for the amplification of mRNA expression. The sequences of primers are as follows: for PAPSS2 (forward primer, 5′-TGTAAAACGACGGCCAGT-3′; reverse primer, 5′-CAGGAAACAGCTATGACC-3′), the α1 chain of collagen type I (Col1, forward primer, 5′-CACCCTCAAGAGCCTGAGTC-3′; reverse primer, 5′-GCTTCTTTTCCTTGGGGTTC-3′), osteopontin (OPN, forward primer, 5′-TCACCATTCGGATGAGTCTG-3′, reverse primer, 5′-ACTTGTGGCTCTGATGTTCC-3′), Smad2 (forward primer, 5′-GAGGAGCAGCTCGCCAA-3′; reverse primer, 5′-CTGTCAAGGTCCGGCCAGCG-3′), Smad3 (forward primer, 5′-TG GTCCTCTGGGCATCTCAGGC-3′; reverse primer, 5′-GGTGAACCTGCTGTTGCCCTCA-3′, GAPDH (forward primer, 5′-ACCACAGTCCATGCCATCAC-3′; reverse primer, 5′-TCCACCACCCTGTTGCTGTA-3′). PCR-amplified products were separated by 1.2% agarose gel electrophoresis. To estimate the degree of accuracy of each transcript, relevant bands were quantified using the NIH Image J software, and the resulting products were normalized against GAPDH levels. For real-time PCR, specific primers for PAPSS2 were synthesized as shown above. Real-time PCR was performed on an ABI PRISM 7500 sequence detection system with SYBR GREEN PCR Master Mix (Applied Biosystems) according to the manufacturer's instructions. The PCR conditions were as follows: 94°C for 1 min followed by 35 cycles of 95°C for 30 s and 58°C for 40 s. All of the reactions were run in triplicate and normalized to the housekeeping gene GAPDH. The evaluation of relative differences of PCR results was calculated using the comparative cycle threshold method.

### Papss2 shRNA lentivirus packaging, tittering and cell infection

To determine the role of PAPSS2 gene in osteoblast differentiation and osteoblastic signaling pathways, we used lentivirus vector mediated PAPSS2 shRNA (Open Biosystems) for packaging PAPSS2 recombinant lentivirus. It was used in accordance with the manufacturer's instructions. Briefly, the 293FT producer cell line was co-transfected with the expression constructs (pLenti-scrambled shRNA, pLenti-P2a, or pLenti-P2b) and packaging mixture. The viral supernatant was harvested after 48–72 hours and titers were determined by infecting HeLa or 293T cells with serial dilutions of concentrated lentivirus. After 48 hours of incubation, the cells were analyzed by RT-PCR, Western blotting, and immunostaining to determine silent efficiency of the PAPSS2 gene. For osteoblast differentiation, the MC3T3 cells were infected with viral supernatant plus 6 µ/ml polybrene (Sigma) for 24 hours were induced with OS media for the indicated times based on different experiments.

### Construction of overexpression vectors, and production of recombinant retrovirus

Retroviral vector pBMP-PAPSS2 was constructed by inserting a full-length 3.64 kb PAPSS2 cDNA (access no. NM_011864) into the EcoRI and Not I site of pBMN-I-GFP (Addgene), and packaging was performed as the protocol from Dr. Garry Nolan Laboratory, Stanford University. Briefly, retrovirus vectors pBMN-I-GFP (control vector) and pBMN-PAPSS2 were separately transfected into the Phoenix-eco tropic packaging cells by CaCl_2_ precipitation method. Following the transfection, the cells were placed in a 32°C humidified incubator for 48 hours (32°C aids in stabilizing the virus). The media containing infectious virus was harvested and filtered through a 0.45 mm filter for titration assay and infecting MC3T3-E1 clone 4 cells. GFP and PAPSS2 protein expression were confirmed by observation of GFP^+^ cells, immunostaining, and Western blot analysis. The retroviruses carrying pBMP-PAPSS2 and pBMN-I-GFP were then used to infect 70–80% subconfluent MC3T3-E1 clone 4 cells in the presence of 6 μg/ml Polybrene.

### Alkaline phosphatase activity measurement

ALP activity was measured as previously described using a p-nitrophenyl phosphate substrate and an incubation temperature of 37°C by a method modified from that of Lowry et al. [41]. In brief, the assay mixtures contained 0.1 M of 2-amino-2-methyl-1-propanol, 1 mM of MgCl_2_, 8 mM of p-nitrophenyl phosphate disodium (Sigma), and cell homogenates. After 3 minutes of incubation, the reaction was stopped with 0.1 N NaOH and the absorbance was read at 405 nm. A standard curve was prepared with p-nitrophenol (Sigma). Each value was normalized with the value in DNA content. DNA in pellets was measured according to the method of Schneider [42].

### Alizarin red staining and von Kossa staining

To measure extracellular matrix Ca deposits for bone nodule formation, cellular matrix was stained using Alizarin red dye which combines with Ca in the matrix as previously described [43], [44]. At 14 days, cells were washed with PBS twice and then fixed with 2.0% formaldehyde. The cells were stained with 40 mmol/L of Alizarin red solution (pH 4.4) for 40 minutes at room temperature and rinsed with deionized water twice. The images of stained cells were captured using a phase contrast microscope with a digital camera (IM50, Leica, Germany). The Alizarin red solution was removed by aspiration, and the cells were rinsed five times with deionized water. The water was removed by aspiration, and the cells were incubated in PBS for 15 min at room temperature on an orbital rotator. The concentration of Alizarin red staining in the samples was determined by comparing the absorbance values with those obtained from Alizarin red standards. The cells were destained for 15 min with 10% (w/v) cetylpyridinium chloride(sigma) in 10 mM sodium phosphate (pH 7.0). The extracted stain was then transferred to a 96-well plate, and the absorbance at 562 nm was measured using spectrophotometer. The mineralization values were normalized to the relative number of PLB as determined directly in the 96-well plates [45]. Because Ca coprecipitates with the phosphate ion in the matrix, von Kossa staining (which stains phosphate ions) was also used for the determination of mineralization in the cultures [46]. Cells were washed with PBS and fixed with 2.0% formaldehyde for 15 minutes. After washing with deionized water 3 times, cells were incubated with 3% silver nitrate at room temperature under ultraviolet light for 1 hour. After washing with deionized water, the images of the stained cells were photographed using phase-contrast microscope.

### Immunostaining

The cells were stimulated with PAPSS2 for the indicated times, plated on 24 well plates and incubated overnight. The cells were sequentially fixed with 100% methanol for 10 minutes, blocked with 10% normal rabbit or mouse serum in PBS for 60 minutes, then correspondingly incubated in mouse anti-PAPSS2 antibody (1 µg/ml, Sigma) rabbit anti-type I collagen (Col I) specific polyclonal antibody, rabbit anti-osteopontin (OPN) polyclonal antibody (1 µg/ml, Invitrogen), and mouse anti-phospho-Smad 2/3 antibody (1 µg/ml, Santa Cruz Biotechnology) in TBS containing 1.5% normal rabbit or goat serum, respectively, for 60 minutes. Cells were then incubated in FITC- or HRP-conjugated anti-rabbit, or mouse IgG (1 µg/ml, Santa Cruz Biotechnology) with 1.5% normal rabbit, donkey, or goat serum, respectively, for 60 minutes. Cells were treated with FITC-conjugated antibody, then washed three times in PBS, and examined with microscopes [47]. The cell nuclei were stained using DAPI to facilitate determination of the number of cells.

### Western blot analysis

Cells were lysed with NP40 buffer (1%NP-40, 0.15 M NaCl, 50 mM Tris, pH 8.0) containing protease inhibitors (Invitrogen). Cell lysates were centrifuged at 12,000 g for 20 minutes at 4°C, and the supernatants were stored at −80°C. Protein quantitation was performed using a BCA protein assay reagent (Pierce, Rockford, IL, U.S.). Equal amounts of protein were denatured in SDS sample buffer and separated on 10% polyacrylamide-SDS gel. Proteins were transferred in 25 mM of Tris, 192 mM of glycine, and 20% methanol to produce polyvinylidene difluoride. Blots were blocked with Tris-buffered saline (TBS; 20 mM of Tris-HCl [pH 7.5] and 137 mM of NaCl) plus 0.1% Tween20 containing 3% dried milk powder. We used anti-phospho-Smad 2/3 antibody (Santa Cruz Biotechnology, CA, U.S.) and anti-PAPSS2 antibody (Abnova, Taiwan) to detect the signals. The antigen-antibody complexes were visualized using the appropriate secondary antibodies (Sigma) and an enhanced chemiluminescence detection system, as recommended by the manufacturer.

### Statistical analyses

Statistical analysis was performed using SPSS-17.0. Data were analyzed using one-way analysis of variance [48]. The Tukey HSD test was used as a post hoc test. Statistical significance across two groups was assessed using the Student's t-test. Differences were considered significant was at *P*<0.05.
